# Supplementary material for: ACD: Antimicrobial chemotherapeutics database
Source: PLoS One. 2020 Jun 25;15(6):e0235193. doi: 10.1371/journal.pone.0235193 (PMC7316249; doi:10.1371/journal.pone.0235193)
Supplement: Attachment — Submitted filename: PONE-D-20-12977_PloseONE.docx [file pone.0235193.s001.docx]

**Manuscript Title: ACD: Antimicrobial chemotherapeutics database**

**Manuscript Number: PONE-D-20-12977**

The ACD database is required as it clubs the properties of many important and significant databases from the prospect of antibacterial resistance.

**The manuscript may be accepted with minor changes.**

1. Slight syntax errors like ….”time to react  ”…. there should be no space after react.; second line of second paragraph, font is different (antibiotic resistance); full form of ECDC should be given;
2. …..and very few are synthetic….This line in the abstract may be rewritten with better sentence format.
3. There should be the same format for heading. Most of them are in Capital sentences while a few are in Capital Word like **Data Collection**. There should be a uniformity in the entire manuscript.
4. As per 2014 WHO report…….updated data of MDR-TB should be provided. Data of 2014 is quite old for a database.
5. Tha abstract may be re-written as it is showing high similarity with the author's web page (amdr.amu.ac.in).
6. Introduction may be elaborated with more emphasis on the need of this database.
7. Comparison of available databases may be discussed in more detail. Further, compare this database with other available databases. Although the ACD database provides more information than current databases, a detail is required on the syntax of the database. Tools used to create ACD may be discussed with other databases and compare the limits and advantages of this database with other similar databases.
8. Its an excellent database so its appication and competativeness of the server should also be highlighted.
