# Supplementary material for: ACD: Antimicrobial chemotherapeutics database
Source: PLoS One. 2020 Jun 25;15(6):e0235193. doi: 10.1371/journal.pone.0235193 (PMC7316249; doi:10.1371/journal.pone.0235193)
Supplement: Attachment — Submitted filename: plos response .docx [file pone.0235193.s002.docx]

**Justification of reviewers’ comments**

**Note: all changes are yellow highlighted**

Reviewr 1: The ACD database is required as it clubs the properties of many important and significant databases from the prospect of antibacterial resistance.

**The manuscript may be accepted with minor changes.**

1. Slight syntax errors like ….”time to react  ”…. there should be no space after react.; second line of second paragraph, font is different (antibiotic resistance); full form of ECDC should be given;

**Compliance:** Remove space after react

"antibiotic resistance" is written in another format instead Times New Roman

ECDC: European Centre For Disease Prevention and Control

2. …..and very few are synthetic….This line in the abstract may be rewritten with better sentence format.

**Compliance:** ….. as per suggestion modified “Antimicrobial chemotherapeutics database (ACD) is comprised of ~4100 synthetic antimicrobial compounds as well as ~1030 active antimicrobial peptides. The Antimicrobial peptides are mainly from biological sources and some of them are synthetic in nature.”

3. There should be the same format for heading. Most of them are in Capital sentences while a few are in Capital Word like **Data**

**Collection**. There should be a uniformity in the entire manuscript.

**Compliance:**: modified as suggested by reviewr.

4. As per 2014 WHO report…….updated data of MDR-TB should be provided. Data of 2014 is quite old for a database.

**Compliance:** recent data of WHO was updated and an additional reference was also included in the text as below.

“treatment [4]. As per WHO MDR-TB factsheet 2018 report about 558,000 new cases of multi-drug resistance tuberculosis (MDR-TB) have been estimated in 2017 and 161,000 cases of MDR or RR TB detected and reported in 2017 [5]. The MRSA (Methicillin-resistant *Staphylococcus aureus*) infected people are 64% more likely to die than the people infected with non-resistant strains as per the WHO report [4].”

5. Tha abstract may be re-written as it is showing high similarity with the author's web page (amdr.amu.ac.in).

**Compliance:** abstract is modified as suggested by the reviewer

6. Introduction may be elaborated with more emphasis on the need of this database.

**Compliance:** Introduction is modified as suggested by the reviewer

7. Comparison of available databases may be discussed in more detail. Further, compare this database with other available databases. Although the ACD database provides more information than current databases, a detail is required on the syntax of the database. Tools used to create ACD may be discussed with other databases and compare the limits and advantages of this database with other similar databases.

**Compliance:** some additional advantages of the ACD database were didcussed along with tools used to attain the objectives.

8. Its an excellent database so its appication and competativeness of the server should also be highlighted.

**Compliance:** Introduction and conclusion section is modified accordingly.

Reviewer #2: In the present database, "ACD: Antimicrobial chemotherapeutics database" author have collected huge informatios about antimicrobial compounds, and compiled it in this database. I my view it will be very useful for researcher working in the field of antimicrobial drug resistance. I have tried this database with two compounds and found positive results. I recommend this dataset for publication in PLOS ONE after two minor suggestions (If possible)
1.Add an option of search compound based on .SDF file of compound (if possible)
2.Add an option to search based on the peptide sequence (if possible)

**Compliance:** I agree with the wornderful suggestion of reviewer but due to lock down no lab is allowed to open, students are not allowed to visit labs. It may take some time to add these featrures. But that may be added even after the study publishd. any way we keep updateing the daatbase. I assure reviewer that these features will be added later. Any way data base will remain active and functional to suffise the need of resaerchers.
